# Artificial Intelligence Frameworks to Detect and Investigate the Pathophysiology of Spaceflight Associated Neuro-Ocular Syndrome (SANS)

**DOI:** 10.3390/brainsci13081148

**Published:** 2023-07-30

**Authors:** Joshua Ong, Ethan Waisberg, Mouayad Masalkhi, Sharif Amit Kamran, Kemper Lowry, Prithul Sarker, Nasif Zaman, Phani Paladugu, Alireza Tavakkoli, Andrew G. Lee

**Affiliations:** 1 Department of Ophthalmology and Visual Sciences, University of Michigan Kellogg Eye Center, Ann Arbor, MI 48105, USA; 2 University of Cambridge, Cambridge CB2 1TN, UK; 3 University College Dublin School of Medicine, Belfield, Dublin 4, Ireland; 4 Human-Machine Perception Laboratory, Department of Computer Science and Engineering, University of Nevada, Reno, NV 89512, USA; 5 Yale University, New Haven, CT 06520, USA; 6 Brigham and Women’s Hospital, Harvard Medical School, Boston, MA 02115, USA; 7 Sidney Kimmel Medical College, Thomas Jefferson University, Philadelphia, PA 19107, USA; 8 Center for Space Medicine, Baylor College of Medicine, Houston, TX 77030, USA; 9 Department of Ophthalmology, Blanton Eye Institute, Houston Methodist Hospital, Houston, TX 77030, USA; 10 The Houston Methodist Research Institute, Houston Methodist Hospital, Houston, TX 77030, USA; 11 Departments of Ophthalmology, Neurology, and Neurosurgery, Weill Cornell Medicine, New York, NY 10065, USA; 12 Department of Ophthalmology, University of Texas Medical Branch, Galveston, TX 77555, USA; 13 University of Texas MD Anderson Cancer Center, Houston, TX 77030, USA; 14 Texas A&M College of Medicine, Bryan, TX 77030, USA; 15 Department of Ophthalmology, The University of Iowa Hospitals and Clinics, Iowa City, IA 50010, USA

**Keywords:** spaceflight associated neuro-ocular syndrome, artificial intelligence, machine learning, microgravity

## Abstract

Spaceflight associated neuro-ocular syndrome (SANS) is a unique phenomenon that has been observed in astronauts who have undergone long-duration spaceflight (LDSF). The syndrome is characterized by distinct imaging and clinical findings including optic disc edema, hyperopic refractive shift, posterior globe flattening, and choroidal folds. SANS serves a large barrier to planetary spaceflight such as a mission to Mars and has been noted by the National Aeronautics and Space Administration (NASA) as a high risk based on its likelihood to occur and its severity to human health and mission performance. While it is a large barrier to future spaceflight, the underlying etiology of SANS is not well understood. Current ophthalmic imaging onboard the International Space Station (ISS) has provided further insights into SANS. However, the spaceflight environment presents with unique challenges and limitations to further understand this microgravity-induced phenomenon. The advent of artificial intelligence (AI) has revolutionized the field of imaging in ophthalmology, particularly in detection and monitoring. In this manuscript, we describe the current hypothesized pathophysiology of SANS and the medical diagnostic limitations during spaceflight to further understand its pathogenesis. We then introduce and describe various AI frameworks that can be applied to ophthalmic imaging onboard the ISS to further understand SANS including supervised/unsupervised learning, generative adversarial networks, and transfer learning. We conclude by describing current research in this area to further understand SANS with the goal of enabling deeper insights into SANS and safer spaceflight for future missions.

## 1. Introduction

Spaceflight-associated neuro-ocular syndrome (SANS) describes a specific collection of neuro-ophthalmic changes that are observed in astronauts in long-duration spaceflight (LDSF) missions [1]. These findings include optic disc edema (ODE), globe flattening, choroidal folds, and hyperopic shift. SANS is one of the largest physiological barriers for planetary spaceflight with the National Aeronautics and Space Administration (NASA) designating it as a “red” risk based on occurrence likelihood and impact on astronaut health and mission performance [2]. With this designation, there is a high priority to further understand SANS pathogenesis and development of countermeasures for future spaceflight. While it is a top priority for mitigation development and a large barrier to future spaceflight, the pathogenesis of SANS is not well understood [1]. The spaceflight environment holds a myriad of limitations, including limited medical and diagnostic capabilities. This austere environment holds various constraints to investigating SANS pathophysiology and subsequent insights into effective countermeasure research. The advent of artificial intelligence (AI) has revolutionized the field of medicine in diverse aspects including disease detection and management [3,4]. The utilization of AI has potential to address several limitations in the spaceflight environment. In this manuscript, we discuss advances in artificial intelligence applications that can be utilized to detect and study the pathophysiology of SANS during spaceflight. To provide context for how AI architectures can conduct these tasks, we first review SANS findings and proposed hypotheses of pathogenesis including cephalad fluid shifts, ocular glymphatic system dysfunction, and genetic factors. Following this overview, we discuss the current limitations in the spaceflight environment to further characterize SANS and its pathogenesis. Subsequently, we provide an overview of key AI techniques that have the potential to address these barriers, including supervised learning, unsupervised learning, generative adversarial networks, and transfer learning. We also provide architectures designed for the further understanding of SANS with the current imaging modalities available on the International Space Station (ISS).

## 2. Spaceflight-Associated Neuro-Ocular Syndrome (SANS)

Vision is of the utmost importance in astronaut health and mission performance during spaceflight missions. Beginning in 1989, astronauts were asked about visual changes following spaceflight missions, which led to multiple reports of visual changes [5]. Persistent anecdotes of visual changes led to further investigation by NASA, including ophthalmic imaging. These investigations led to the first description of SANS by Mader et al., which examined the various ophthalmic changes recorded in astronauts following spaceflight [5]. Employing a combination of modalities and procedures including optical coherence tomography (OCT), fundus examination, magnetic resonance imaging (MRI), and lumbar puncture, this report identified several core changes, including optic disc edema, posterior globe flattening, retinal nerve fiber layer thickening, cotton wool spots (CWS), and hyperopic refractive shifts following LDSF [5]. Mader et al. explored several etiologies to explain their findings, including localized fluid shifts as the pathogenesis, citing the delicately balanced pathways of flow through the intracranial subarachnoid space (SAS) and the optic nerve SAS. If microgravity altered the flow in these routes, it could impede outflow and cause a buildup of CSF in the optic nerve sheath (ONS). This hypothesis for SANS pathogenesis has since been explored in-depth and will be discussed at length in the subsequent section.

### Proposed Pathophysiology of SANS

Knowledge of SANS and its pathophysiology is constantly evolving. The mechanisms underlying the development of SANS continues to be an area of investigation, and, in line with the multiple manifestations of its presentation, its etiology is likely multifactorial [6,7,8]. This section’s review of proposed pathophysiology for SANS is intended to provide context for subsequent sections reviewing the utilization of AI applications to further understand SANS.

“Spaceflight-associated neuro-ocular syndrome” was initially termed “Vision Impairment and Intracranial Pressure” (VIIP) because it was thought that elevated intracranial pressure (ICP) was the driving factor of SANS [1,2]. It has been hypothesized that cephalad fluid shifts observed during microgravity may lead to elevated ICP [9,10]. Under terrestrial gravitational conditions in the standing position, fluid at rest in the body (with a particular focus towards intravascular and cerebrospinal fluid) exerts a hydrostatic pressure downwards [9,10,11]. Weightlessness causes this pressure to decrease (P = ρgh where ρ = density, g = gravity, h = height, and there is a reduction in g due to the weightless environment), leading to a fluid shift towards the head. Microgravity has been observed to cause stagnation and even reversal in the internal jugular veins (IJVs) of an LDSF crew [12]. If similar shifts occur in the ocular veins, their congestion could spur choroidal expansion and CWS formation. Cephalad fluid shifts are thought to impose similar restrictions on cerebral spinal fluid (CSF) flow and drainage, a blockage that poses particular risk to the choroid and optic nerve. In normal physiology, CSF drains from the choroid plexus into the cerebral vasculature, flowing through the brain and ocular system. A loss in hydrostatic pressure may slow CSF flow velocity and decrease its resorption [13], overwhelming decompression mechanisms. The accumulated CSF may cause swelling and elevated pressure in the optic nerve sheath, which in turn contributes to ODE and globe flattening [5,14]. In the setting of presumed elevated ICP, VIIP shared similarities with terrestrial idiopathic intracranial hypertension (IIH), primarily ODE in the setting of elevated ICP. Nevertheless, it is worth noting that IIH is additionally distinguished by the occurrence of persistent headaches, double vision (diplopia), and pulsating ringing in the ears (pulsatile tinnitus), which are clinical aspects that have not been extensively documented/reported among SANS astronauts. Moreover, the SANS condition is characterized by the presence of asymmetric or unilateral ODE, while IIH typically manifests with bilateral ODE [1,15]. Furthermore, opening pressures on post-flight lumbar punctures in astronauts with SANS revealed only mildly elevated postflight ICP of a significant value observed in terrestrial IIH, although no lumbar punctures have been performed during spaceflight to date. [1,5].

Another discrepancy that has also been noted is that if venous pressures were to cause elevated ICP is that intraocular pressure (IOP) should be elevated as well [16]. Persistent IOP elevation during LDSF has not been observed [16]. In addition, central venous pressure (CVP) has not been elevated following the observations of multiple missions [17]. To help uncover and explain these observations, the effects of weightlessness on tissue pressures have been proposed and investigated in its role in SANS pathogenesis [16,17,18]. An interesting observation is that there is a positive relationship between individuals developing SANS and pre-flight body weight [19], while in microgravity, there is a reduction in tissue compressive forces [16]. The effect of the removal of the overall tissue compressive forces has been observed to be greater than the weight of an individual [19,20]. This reduction of tissue compression leads to reduced venous pressure, and this effect is more pronounced in individuals of larger weight. The reduction in venous pressure leads to a reduction in transmural pressure [16]. Given that there are no typical postural and diurnal alterations to CVP during spaceflight, this change is more persistent in the microgravity environment. The continued transmural pressure during LDSF may lead to remodeling of the eye over time, which may lead to signs of SANS in astronauts [16].

In the Studying the Physiological and Anatomical Cerebral Effects of CO_2_ and Tilt (SPACECOT) study, cerebral blood volume (CBV) was analyzed with near-infrared spectroscopy (NIRS) in subjects undergoing head-down tilt, a terrestrial analog for SANS [21]. The results from the SPACECOT study observed an increase in CBV in individuals undergoing HDT. The authors in this study hypothesized that persistent increased CBV pulsatility during LDSF may lead to remodeling of ocular structures [21]. The remodeling process helps to explain why astronauts continue to have persistent SANS findings even after returning to Earth [21].

Mechanical shifting of the brain in the upwards direction has been proposed as a contributor to SANS through its subsequent uplifting of the optic chiasm. Evidence of an upward shift of the brain and its effects was observed by Roberts et al. in a study comparing pre- and post-flight MRI images of 34 astronauts’ brains [22]. The authors examined anatomical changes in the brain and its CSF spaces, tracking vertical brain displacement, central sulcus volume, ventricle volume, and changes in the volume of CSF spaces at the vertex. Astronauts were grouped based on mission length, resulting in 18 LDSF and 16 short-duration (SD). High-resolution cine MRI clips were generated for 12 astronauts within the LDSF group and 6 in the SD group. In these clips, upward shift of the brain was observed after all LDSF but none of the SD flights. Three of the twelve long-duration astronauts developed ODE; all three of these subjects also exhibited irregular CSF pressure and narrowing of the central sulcus. However, these symptoms also appeared in several other members of the LDSF group who did not develop ODE, indicating that alterations in CSF pressure may not levy sufficiently robust effects to cause ODE. Narrowed CSF spaces, including the central sulcus, occurred often within the LDSF group and were correlated with upward brain shifting. A reduction in volume in these CSF spaces may lead to subsequent ventricle congestion [22]. These findings are valuable in light of a mathematical model of ONS mechanics composed by Shinojima et al. Their model used optic nerve sheath diameter (ONSD) to calculate CSF pressure, enabling them to monitor CSF pressure in astronauts using only measures of their ONSD. However, during application, the model reported implausibly high CSF pressures for several astronauts [23]. This result led the authors to believe that the astronauts’ ONSs had elasticities that differed from the standard measure used in the model. They hypothesized that this degraded elasticity was an effect of the upward shift of the brain, whose consistent backwards pull on the optic nerve could deform the ONS dura. This model reinforces Roberts et al.’s findings, and the studies in conversation with each other lend support to upward brain shifting as a potential mechanism for SANS [23].

Recent findings have tentatively linked SANS to the ocular glymphatic system. The glymphatic system is a recently discovered network of perivascular pathways within the brain that washes out excess interstitial fluid, clears waste from the brain and central nervous system, and distributes compounds such as lipids, glucose, and amino acids [24]. It has also been proposed as a primary mechanism for CSF transport [25]. In this model, CSF flows into the cranial SAS from the choroid plexus, traveling through the brain via perivascular spaces (PVS). It eventually enters the complex brain parenchyma, flushing out waste and interstitial fluid and then draining into the lymphatic system. Cranial glymphatic flow was linked to the ocular system in a 2017 study by Mathieu et al. which observed CSF inflow into the optic nerve spaces [26]. Additionally, it has been illustrated that ocular fluids are cleared from the eye via perivenous drainage pathways in rodents [27]. This connection led to the definition of a specialized ocular glymphatic system, one which relies on a delicate balance of pressures, polarization, and venous and arterial functionality. Wostyn et al. discusses the potential mechanisms and consequences of ocular glymphatic dysregulation, focusing specifically on instances of PVS dilation [25]. They identify two key mechanisms by which spaceflight could induce PVS dilation. The first draws from Marshall-Goebel et al.’s IJV findings, described above. The altered hemodynamics that they describe cause cerebral vein distention. Because an increase in venous volume would necessarily shrink the surrounding perivenous outflow, this vascular congestion could halt glymphatic outflow and drainage. CSF continuing to enter via the periarterial spaces would be unable to move forwards and instead accumulate, causing the observed periarterial dilation. Wostyn et al. further propose that weakened CSF resorption, when combined with the impaired outflow, can cause CSF buildup along the ONS, generating pressure near the optic nerve head and contributing to globe flattening and ODE. This buildup may also contribute to choroidal folds by forcing choroid expansion, increasing its rigidity, and predisposing it to corrugation [28]. Further research in the ocular glymphatic system during microgravity may provide additional insights into SANS.

Along with the physical mechanisms proposed to drive SANS, research suggests that genetics and vitamin status may predispose some astronauts to develop SANS [29,30]. Zwart et al. investigated whether astronauts with naturally higher levels of 1-carbon metabolites experienced SANS-associated ophthalmic changes [29,30]. 1-carbon metabolite concentration was shown to be correlated with ophthalmic change in several astronauts in 2012 [31]. The authors investigated five single-nucleotide polymorphisms (SNPs) within several genes involved in 1-carbon metabolism to assess their potential correlation with these symptoms: globe flattening, choroidal folds, optic disc edema, CWS, and change in diopters. They created three models of association, which tracked the development of SANS against (1) days in space, (2) days + presence of polymorphisms, and (3) days + presence + vitamin B levels. Comparing these models revealed several genetic associations. The MTRR 66 G allele was highly correlated with choroidal folds and CWS; astronauts homozygous for the GG genotype always presented both symptoms, while those homozygous for AA developed none. The SHMT 1 C allele was correlated with ODE, whereas the SHMT 1420 TT allele appeared to provide protection from it as none of the astronauts with the TT allele developed ODE. Although none of the five SNPs were associated with diopters or globe flattening, these findings nonetheless provide strong support for the possibility of genetic predisposition for SANS. Building on their previous work, Zwart et al. published a 2019 study investigating proposed links between 1-carbon metabolic polymorphisms and ODE. The study’s subjects experienced head-down tilt bed rest (HDTBR), a terrestrial analog technique that mimics the hydrostatic pressure of microgravity [32], under 0.5% elevated CO_2_ levels for 30 days. To determine the risk of developing ODE, total retinal thickness (TRT) and RNFL thickness were recorded using OCT. Changes in thickness were then analyzed against the participants’ allele types and vitamin B levels. The authors found that TRT in participants with 3–4 risk alleles increased dramatically more than in those with 0–2, creating a difference of 40 μm. Furthermore, pre-HDTBR TRT for participants with 3–4 risk alleles was 14 μm greater than the low-risk group at baseline. Thus, the risk alleles were both correlated with larger TRT at onset and greater response during exposure to adverse conditions. These findings are striking and offer an intriguing explanation of the differential development of SANS in astronauts (Table 1).

## 3. Medical Diagnostic Challenges during Spaceflight

The field of space medicine is faced with distinctive challenges in the diagnosis and treatment of medical conditions during spaceflight [35]. These challenges can be attributed to several factors, including constrained medical capabilities, a scarcity of medical personnel, and restricted access to diagnostic imaging [1,2,36]. Furthermore, the absence of invasive approaches such as fluorescein angiography and lumbar punctures, along with imaging modalities such as MRI, may present as an obstacle in understanding particular medical conditions such as SANS [37]. The comprehensive documentation of SANS requires careful evaluation of multiple imaging modalities. This presents a significant challenge for astronauts because the available diagnostic imaging capabilities are limited, and the transmission of high-quality images can be delayed, particularly when traveling away from Earth [37]. In addition, the limited number of astronauts participating in space missions poses a challenge in obtaining a comprehensive dataset for grasping SANS, as well as for the purposes of developing artificial intelligence (AI) models for diagnostic purposes [36]. In this section, we describe the unique challenges that are experienced during spaceflight.

Despite these challenges, recent advances in AI have shown promising results in the diagnosis of SANS [38]. Various advances have been made for terrestrial ophthalmic diseases including IDx-DR, the first FDA-approved AI device for the diagnosis of diabetic retinopathy with fundus imaging [39]. As fundus imaging is available on the ISS, such technology can be relatively quickly translated and applied to SANS. In addition to fundus imaging, OCT and orbital ultrasound are accessible onboard the International Space Station (ISS) and can provide valuable information for the diagnosis of SANS [40]. As seen with fundus imaging, OCT imaging is also well-suited for AI-based automated diagnosis due to the ease of acquiring high-quality images [41]. The interpretation of various imaging modalities may be aided by AI-based automated diagnostics, providing prompt monitoring of SANS.

### 3.1. Delayed Communication and Imaging Transmission

One of the anticipated obstacles in the diagnosis of medical conditions during planetary spaceflight involves the delayed transmission of high-quality medical images, particularly when the spacecraft is travelling away from Earth [36,42]. This can be attributed to several factors, such as limited bandwidth, distance, and communication interruptions [36]. In various cases, including SANS documentation, medical images need to be taken onboard the spacecraft, which can be sent to Earth for diagnosis by medical experts.

For planetary travel, this process may take several hours or even days depending on various factors including the distance between the spacecraft and Earth and the size/quality of images. This delay in transmission can be critical when countermeasure initiation is imperative. To overcome these challenges, several approaches have been proposed, including the use of advanced imaging techniques, automated analysis algorithms, and even AI-based diagnosis/monitoring that we discuss in subsequent sections [3,43].

There have been several advancements in onboard medical imaging technologies that can provide high-quality medical images without the need for transmission to Earth. For instance, researchers have developed a portable ultrasound device that can be used onboard the International Space Station (ISS) to provide real-time imaging of the heart, blood vessels, and other organs [44,45,46]. Such systems can also be useful in diagnosing/monitoring SANS, which is a major concern for LDSF missions.

### 3.2. Limited Individuals Undergoing Spaceflight

Another significant challenge in diagnosing medical conditions during spaceflight is the limited number of astronauts, making it difficult to have a broad dataset for understanding SANS. Since SANS is a relatively new condition, there is limited data available on its causes, mechanisms, and long-term effects [5]. Moreover, the limited number of astronauts who have developed SANS makes it difficult to identify the risk factors associated with the condition [5,47]. The small proportion of astronauts who suffer SANS makes it difficult to completely comprehend this condition. Few astronauts travel over extended periods of time in space, and not all of them encounter SANS. The difficulty of defining SANS is exacerbated by the variation in the onset and severity of the illness in various people [47]. The inability to properly comprehend the pathophysiology of the illness is further hampered by the absence of a comprehensive dataset on SANS.

Data for SANS primarily comes from astronauts who have completed space missions [47,48]. These investigations have been invaluable regarding new information about the risk factors, clinical characteristics, and prevalence of SANS. However, these studies are constrained by small sample sizes and reliance on information gathered from numerous missions spanning many years. Data collected during these missions may not be uniform because in-flight medical technology continues to change (e.g., optical coherence tomography angiography capabilities onboard the ISS in 2018).

One potential solution to this challenge is to collect additional data on astronauts who have spent long durations in space. This can be achieved through longitudinal studies that follow astronauts before, during, and after their space missions to monitor any changes in their visual and neural health. Furthermore, the development of wearable technologies that can continuously monitor astronaut health during spaceflight can provide real-time data on any changes in visual and neural health, including the onset of SANS [49,50,51].

## 4. Imaging Onboard the International Space Station

There are several types of retinal imaging techniques that can be used onboard the ISS to aid in the diagnosis and monitoring of ocular abnormalities, including fundus photography, optical coherence tomography (OCT), and orbital ultrasound. Each of these imaging techniques can provide valuable information for diagnosing and monitoring ocular abnormalities in astronauts during spaceflight.

**Figure 1:**
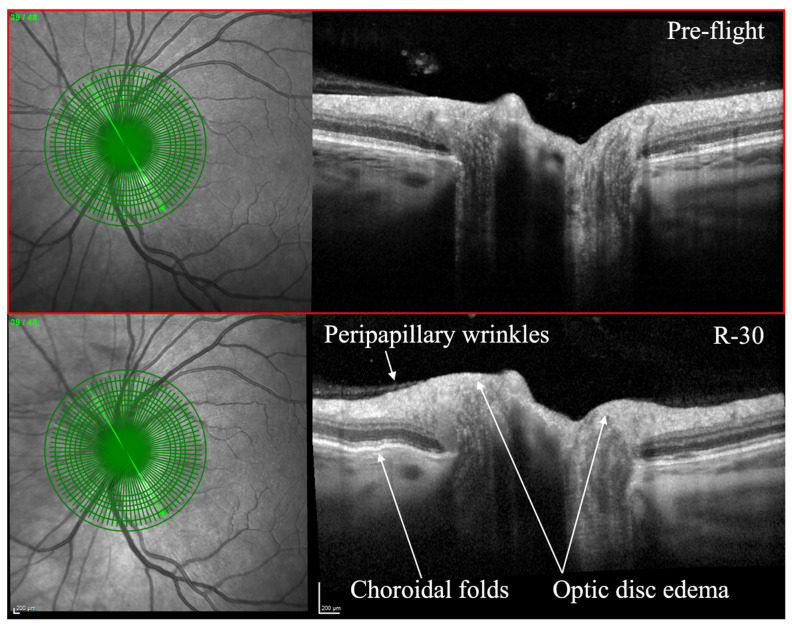
Optical coherence tomography (OCT) of an astronaut’s eye pre-flight (**top image**) and 30 days before to returning to Earth from spaceflight (**bottom image**, R-30). OCT in R-30 demonstrates optic disc edema, choroidal folds, and peripapillary wrinkles. Courtesy of NASA. Reprinted with permission from Ong et al. Spaceflight-associated neuro-ocular syndrome: proposed pathogenesis, terrestrial analogues, and emerging countermeasures. British Journal of Ophthalmology. January 2023. https://doi.org/10.1136/bjo-2022-322892 (accessed on 25 June 2023) under Creative Commons Attribution NonCommercial (CC BY-NC 4.0) license (https://creativecommons.org/licenses/by-nc/4.0/legalcode) (accessed on 25 June 2023).

1. Fundus photography: Fundus photography is a non-invasive imaging modality that captures detailed images of the retina, optic nerve, and blood vessels. This technique can be used to detect and monitor a variety of ocular abnormalities, including diabetic retinopathy, glaucoma, macular degeneration, and retinal detachment [52].
2. Optical coherence tomography (OCT): OCT is an imaging technique that utilizes light waves to produce a detailed, cross-sectional image of the retina. This technique can provide detailed information on the thickness of the retinal layers, which can be useful for diagnosing and monitoring conditions such as macular edema, macular holes, and vitreomacular traction [53,54]. OCT can also be used to monitor changes in the retinal nerve fiber layer thickness, which is important for diagnosing and monitoring glaucoma [55]. OCT has been utilized to evaluate SANS during and after spaceflight (Figure 1) [7]. As of 2018, OCT angiography (OCTA) and MultiColor Imaging became available onboard on the ISS [1]. These recent advances will allow for additional insights into changes within the retina and retinal vasculature during spaceflight.
3. Orbital ultrasound: Orbital ultrasound is a non-invasive imaging technique that employs sound waves to produce images of the structures in and around the eye. This technique can be used to visualize the optic nerve and surrounding tissues, which can be useful for diagnosing and monitoring conditions such as optic nerve swelling (papilledema), retinal detachment, and retrobulbar hematoma [56].

## 5. Introduction to Artificial Intelligence-Based Diagnosis in Ophthalmic Imaging

The prompt identification and management of SANS is imperative to minimize its detrimental impact on the well-being of astronauts. The transfer of high-quality visual information from space to Earth may experience latency issues because of restricted bandwidth, which could delay timely medical assessment and intervention [57]. Recent advancements in AI have the potential to significantly improve the diagnostic capabilities in spaceflight [58]. AI algorithms can process large amounts of medical data quickly and accurately, allowing for the detection of subtle changes in ocular anatomy that could indicate the development of SANS. Additionally, AI can help generate high-quality images from low-resolution or noisy data, which is particularly important when considering the limited diagnostic and imaging capabilities on board spacecrafts [4,59].

One area where AI is already making significant contributions is in the field of automated diagnosis in ocular imaging. Deep learning algorithms have demonstrated high accuracy in detecting diabetic retinopathy, a highly prevalent ocular disorder that can lead to vision loss if untreated [60]. Furthermore, the IDx-DR system, which is the first FDA-approved AI device for the automated diagnosis of diabetic retinopathy [61], has been shown to achieve a diagnostic accuracy rate of 87.4%, which is comparable to practicing ophthalmologists [61]. Similarly, AI-based algorithms can be trained to automatically detect and quantify changes in the retinal anatomy that are indicative of SANS. This can be achieved through both supervised and unsupervised learning techniques. In supervised learning, the machine learning algorithm is trained on a dataset of labeled images [62], with the labels indicating the presence or absence of SANS. The algorithm can then use this knowledge to identify similar patterns in new, unlabeled images [62]. In unsupervised learning, the algorithm learns to identify patterns in the data without any labeled examples and can be used to identify features that are associated with SANS [63].

The success of IDx-DR and other AI-based diagnostic devices highlights the potential of AI in ocular imaging and its ability to overcome some of the challenges associated with spaceflight medical diagnostics. This section will examine the potential application of AI in the automation of the diagnosis of SANS under conditions of transmission delays in high-quality imaging to mission control centers and limited medical personnel during spaceflight.

## 6. Convolutional Neural Networks, Supervised Learning, and Unsupervised Learning for SANS

In this section, we will discuss two primary techniques for artificial intelligence-based diagnosis in ocular imaging that can be utilized for SANS: Supervised and Unsupervised learning. We also discuss convolutional neural networks (CNNs), which serve as a fundamental architecture in deep learning for imaging processing/recognition. It is important to note that many of the applications in the context of spaceflight and SANS have not yet been tested in-flight. The purpose of this section is to discuss foundations of the technology, terrestrial applications, and the connections of its relevance to SANS for potential future applications.

Supervised learning involves training an algorithm using labeled data, where the correct diagnosis or classification of the image is provided to the algorithm [62]. This allows the algorithm to learn how to accurately diagnose new images it has not seen before. Unsupervised learning involves training an algorithm using unlabeled data, where the algorithm must identify patterns and structures within the data itself [63]. This approach is useful when there is not enough labeled data available, or when new patterns may arise that were not previously identified.

With retinal imaging, these two revolutionary techniques may be able to accurately detect and diagnose retinal changes that may be indicative of SANS [64]. Additionally, the use of AI-based diagnosis can help to mitigate the challenges of limited medical staff and diagnostic capabilities during spaceflight, allowing for more efficient and accurate diagnosis of ocular changes. Throughout this section, we will discuss various studies that have explored the use of these techniques in the context of ocular imaging and spaceflight.

### 6.1. Convolutional Neural Networks

Deep learning is a subset of machine learning (ML), which is itself a subset of AI [65]. Unlike traditional programming, in which a set of rules are given to solve a particular problem, machine learning algorithms allow a computer to learn from data inputs and improve over time [65]. One of the most popular types of machine learning algorithms used in deep learning is the convolutional neural network (CNN) [65]. CNNs are a class of neural networks that are commonly used for image classification and recognition [66]. They are largely inspired by the structure and function of the visual cortex in the brain [67]. The architecture of a CNN is typically composed of a series of (1) Convolutional layers, (2) Pooling layers, and (3) Fully connected layers. In a CNN, the input image is passed through multiple convolutional layers. Every convolutional layer applies a set of filters to the original input image to extract different features [67,68]. The filters are learned by the network during training, and they help to identify patterns in the image that are important for classification [68,69]. After the convolutional layers, the output is typically passed through one or more pooling layers, which downsample the features to reduce computational complexity [68,69]. Finally, the output is passed through one or more fully connected layers, which combine the features to produce a classification [67,68,69].

While CNNs are powerful tools for analyzing retinal images, they often require significant amounts of labeled data for training. In the context of spaceflight, where the number of astronauts is limited, acquiring large amounts of labeled data may be challenging. This limitation arises from a variety of factors, encompassing the distinct and rigorous conditions of the space environment, the prolonged durations of missions, and the absence of resupply capabilities. However, recent advances in transfer learning, where pre-trained models are fine-tuned on a smaller dataset, have shown promise in reducing the amount of labeled data required for training [69,70].

**Figure 2:**
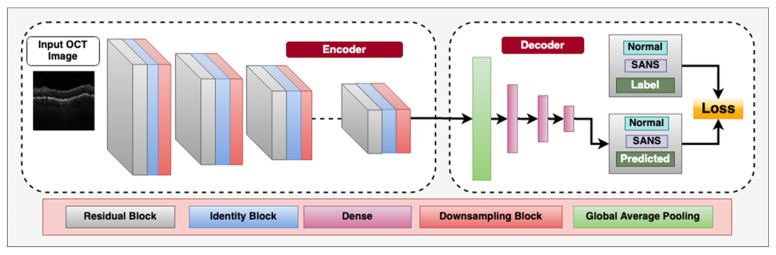
Deep convolutional neural network architecture designed for classifying between spaceflight associated neuro-ocular syndrome (SANS) and non-SANS in optical coherence tomography (OCT) images, an imaging modality onboard the International Space Station (ISS). The encoder consists of residual blocks which have a convolution, batch-normalization, leaky-ReLU activation layer and a residual connection from the input to the output. This is followed by an Identity block, which consists of a convolution layer, batch-normalization layer, and leaky-ReLU layers to learn inherent features. We also utilize a sub-sampling block, which downsamples the spatial features to half the size using stride = 2 convolution operator. The decoder consists of a Global average pooling layer to calculate the channel-wise average of the features and the three dense, fully connected layers for flattening the 2D spatial features to 1D features. The labels utilized are “Non-SANS” and “SANS”, and we utilize supervised cross-entropy loss function to train the model.

Overall, the application of CNNs and other deep learning techniques in retinal imaging holds great potential for the automated diagnosis with ophthalmic imaging during spaceflight missions. Figure 2 demonstrates a CNN architecture that can utilize multiple blocks to classify between OCT images with SANS findings and those that do not have SANS findings.

### 6.2. Similarities and Differences between Supervised and Unsupervised Learning

Supervised and unsupervised learning are two fundamental approaches to machine learning that have been extensively used in various fields, including medical imaging analysis [71]. Both approaches involve training models to identify patterns and make predictions based on input data, but they differ in their training process, the nature of the input data, and the types of tasks they are suitable for.

Supervised learning is a type of machine learning technique that involves training a model on labeled data with the goal to predict the output for new input data [62]. The input data and their corresponding labels are used to guide the learning process and adjust the model’s parameters to minimize the prediction error [62]. In medical imaging analysis, supervised learning has been used for various tasks, including classification of diseases, segmentation of anatomical structures, and detection of abnormalities [62]. Some examples of supervised learning algorithms that have been used in medical imaging analysis include support vector machines (SVM), random forests, decision trees, and deep learning models such as CNNs [68,69].

Unsupervised learning is another type of machine learning technique that involves training a model with unlabeled data to identify hidden patterns or structures in the data [63]. As opposed to supervised learning, unsupervised learning does not require labeled data or the prediction of a specific output [63]. Instead, the model learns from the input data distribution and tries to cluster similar data points or identify outliers [63]. Unsupervised learning has been used in medical imaging analysis for various tasks, such as image registration, data compression, and dimensionality reduction [72,73]. Some examples of unsupervised learning algorithms that have been used in medical imaging analysis include k-means clustering, principal component analysis (PCA), and autoencoders [74,75,76].

Despite their differences, supervised and unsupervised learning share some similarities. For example, both approaches involve training a model to learn from data, which requires a large dataset and computing resources [77]. Both approaches also require careful preprocessing of the input data, including normalization, augmentation, and feature extraction [62,63]. Moreover, both approaches have their advantages and limitations depending on the nature of the tasks and the characteristics of the data.

### 6.3. Supervised Learning for SANS

One of the key advantages of supervised learning is its ability to predict a specific output for novel input data, rendering it suitable for tasks such as classification and segmentation [78]. Supervised learning also allows for the incorporation of prior knowledge and domain expertise into the model through the labeled data [62]. However, supervised learning requires a large amount of labeled data for training, which can be time-consuming and expensive to obtain [62,78,79]. Additionally, supervised learning models are prone to overfitting if the training data is not representative of the test data, and the model’s performance can suffer if the labeled data contained lots of noise or is incomplete [78].

In contrast, unsupervised learning does not require labeled data and can identify hidden patterns or structures in the data, making it suitable for tasks such as clustering and data visualization [74,80]. Unsupervised learning is also more scalable and adaptable than supervised learning because it can learn from unlabeled data and adapt to new data distributions without the need for retraining [63,80]. However, unsupervised learning models are more difficult to evaluate and interpret than supervised learning models because they do not have a specific output to predict [63].

In regard to the applying these techniques to SANS, supervised learning has been applied terrestrially in ophthalmic diagnosis using retinal imaging modalities such as fundus photography and OCT [70]. These modalities enable the visualization of various structures in the retina including blood vessels, the optic nerve head, and the macula [70]. An emerging approach for using supervised learning in ophthalmic diagnosis is through retinal vessel segmentation [81]. Retinal vessel segmentation involves the separation of blood vessels from the surrounding retinal tissue in fundus images [81]. Accurate vessel segmentation is critical for the diagnosis of various retinal diseases because changes in the appearance and morphology of vessels can indicate underlying pathology [81]. Several studies investigated retinal segmentation using different networks and algorithms. For example, Gegundez-Arias et al. presented a deep learning approach utilizing the U-Net network architecture, which integrates residual blocks and batch normalization techniques to effectively segment blood vessels in fundus images [82]. Similarly, Boudegga et al. proposed a U-shaped deep learning network framework capable of accurately segmenting retinal blood vessels [83]. Moreover, a study by Oliveira et al. presented a technique that minimizes the computational complexity of the convolutional layer in a network, while also preserving segmentation accuracy [70]. This was achieved by combining a multi-scale wavelet transform with a multi-scale convolutional neural network to segment retinal blood vessels [70]. Tan et al. introduced a spatial attention mechanism into their new lightweight pyramid network in order to fuse multi-scale features and retain the structural information of retinal vessels. The construction of the pyramid hierarchy model is aimed at producing multi-scale representations, which yielded promising results for better visualizing vessel structures and better understanding the location of vessels [84].

### 6.4. Unsupervised Learning for SANS

Delayed transmission of high-quality imaging data is a challenge that is faced during spaceflight missions [57,63]. This unique spaceflight limitation can be overcome by employing AI to process the images onboard the spacecraft. Unsupervised learning is a machine learning technique that is well suited for processing high-dimensional, complex data such as images without the need for explicit supervision [62,85].

Terrestrially, unsupervised learning has been used to identify hidden patterns or features in data and can be used for tasks such as image reconstruction and denoising [86]. In regard to SANS, unsupervised learning can be used to analyze and interpret fundus and OCT images, without the need for explicit labeling [63]. One study used an unsupervised learning technique known as a deep Boltzmann machine to extract features from fundus images for the purpose of classifying different stages of diabetic retinopathy [87]. Another study used a deep autoencoder, which is a type of unsupervised learning algorithm, to learn the features of OCT images for the purpose of classifying glaucoma.

Image reconstruction in unsupervised learning may be a solution to the delayed transmissions during spaceflight for SANS images. Image reconstruction is the process of creating a high-quality image from a set of low-quality or compressed images [88]. Unsupervised learning can be employed for image reconstruction in spaceflight missions where there is limited bandwidth for transmitting high-quality images to Earth [37,88]. High-quality images, such as SANS OCT images, may be compressed and possibly sent and received quicker back on Earth. Image reconstruction may be utilized on Earth to reconstruct these high-quality images for terrestrial imaging experts to evaluate in a timely manner.

## 7. Generative Adversarial Networks

A Generative Adversarial Network (GAN) is a type of AI that is revolutionizing medicine [89]; GANs consist of two neural networks: (1) generator and (2) discriminator [64,90]. The generator network creates new data that is similar to a training dataset, while the discriminator network learns to distinguish between synthesized and real data [64,90]. GANs have been employed in a variety of diverse applications, including image and audio synthesis, data augmentation, and anomaly detection [91]. In the context of spaceflight, GANs have the potential to generate high-quality images from low-resolution or noisy data, as well as to provide training data for machine learning models. In this section, we discuss the foundation of GAN technology and current applications in the terrestrial setting. We then discuss how this emerging technology is relevant to SANS for potential future applications. As a relatively new technology, rigorous validation for space medicine applications will be extensively required in the future prior to in-flight deployment.

GANs have been used extensively for image synthesis tasks, including super-resolution, inpainting, and style transfer [92,93,94]. Super-resolution refers to the task of generating a high-resolution image from a low-resolution input, while inpainting involves filling in missing or corrupted regions of an image [95]. Style transfer refers to the process of transferring the style of one image onto another while preserving the content [96]. These tasks are particularly relevant for spaceflight, where images may be noisy, low-resolution, or corrupted due to transmission delays or hardware limitations.

### 7.1. GANS for Denoising Retinal Imaging and Data Generation

One application of GANs for spaceflight is the generation of high-quality retinal images from low-resolution or “noisy” data. For example, GANs have been used to generate high-resolution fundus images from low-resolution images, with results comparable to those obtained from traditional image processing methods [97,98]. GANs can also be utilized in the field of spaceflight to generate synthetic training data for machine learning models to address limited data during spaceflight. GANs have the potential to produce substantial amounts of synthetic data that exhibit similarities to authentic data, albeit with differences in lighting, orientation, or other parameters [64,99]. The network responsible for generating data is trained to produce synthetic data that can effectively deceive the discriminator network into perceiving it as authentic data [64]. This process continues iteratively until the generator can generate synthetic data of higher quality that cannot be discerned from authentic data [64]. A study by Zhao et al. utilizing GANs and related frameworks in a learning-based approach was able to successfully synthesize composite images of retinal fundus and neuronal structures. The model exhibited the ability to acquire knowledge from limited training sets comprising merely 10 to 20 cases. The images that were synthesized exhibited consistent tubular structures while displaying varying textural characteristics [99].

This ability to synthesize data can be useful for training machine learning models on datasets that are limited in size such as SANS data. For example, GANs have been used to generate synthetic OCT images for monitoring of macular edema post-therapy, with results comparable to those obtained from real data [100]. Ultimately, GANs have the potential to improve the quality and quantity of retinal images obtained during spaceflight, as well as to provide synthetic training data for machine learning models. Future research directions may focus on developing GANs that are robust to noisy or corrupted data, and on integrating GANs into spaceflight hardware and software systems.

### 7.2. GAN Non-Invasive Angiogram Synthesis for SANS

Spaceflight imposes limitations on the availability of medical resources and diagnostic techniques, making it necessary to explore alternative approaches for medical imaging. For example, fluorescein angiography (FA), which is an invasive and nephrotoxic diagnostic test, is a relatively common procedure on Earth, but its risks are exacerbated in the spaceflight environment due to limited medical and safety capabilities [101,102]. While FA has been never been utilized during spaceflight, its utility in understanding retinal vascular changes may provide further insights into SANS pathophysiology. However, recent studies have explored the use of generative adversarial networks (GANs) to generate synthetic angiographic images from fundus photography, a non-invasive imaging modality on the ISS. One study published in 2020 used a deep convolutional GAN to generate synthetic FA images from fundus images. The researchers demonstrated that their GAN model was able to generate high-quality angiographic images that were comparable to those obtained from FA in terms of vessel segmentation and perfusion patterns. The study showed that GAN-based angiographic synthesis has the potential to be a useful tool for diagnosing and monitoring retinal vascular diseases in space.

**Figure 3:**
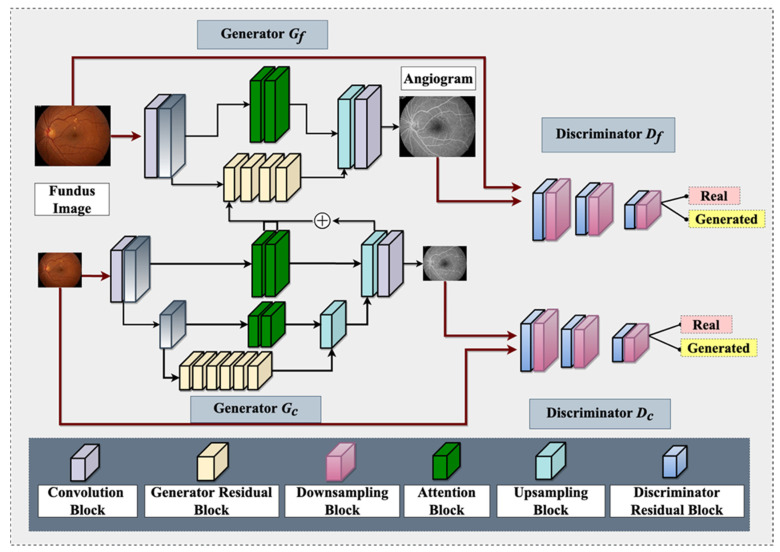
An overview of proposed Fundus-to-Fluorescein Angiography translation generative adversarial network (GAN) architecture to produce SANS angiograms from fundus images. The architecture consists of two generators and two discriminators (for coarse and fine images). Each of the architectures contains distinct blocks, namely: convolution, generator residual, discriminator residual, downsampling, attention, and upsampling. One of the intermediate layers of the coarse generator is added with the Fine generator’s intermediate layer for feature fusion. The generator utilizes reconstruction and adversarial loss, whereas the discriminator utilizes adversarial and feature-matching loss.

It is worth noting that OCTA is now available on the ISS for studying retinal vasculature [1]. However, OCTA has its limitations as an angiographic modality, such as a relatively smaller field of view [103], which can be overcome with GAN-based angiographic synthesis to provide a larger view of the retinal vasculature. This can be especially useful in monitoring the effects of microgravity on the retinal vasculature in space. Figure 3 demonstrates a GAN architecture that can take non-invasive fundus images on the ISS and generate angiograms for retinal vasculature analysis. In summary, the use of GAN-based angiographic synthesis from fundus images has the potential to overcome the limitations of invasive and nephrotoxic tests such as FA and provide a non-invasive and real-time alternative for diagnosing and monitoring retinal vascular diseases during spaceflight

## 8. Transfer Learning

Transfer learning is a technique that involves utilizing pre-existing neural network architectures and weights to address novel and intricate tasks within a particular domain [104]. This approach eliminates the need to build a deep learning model from scratch, or gather a significant amount of data. This is particularly useful for SANS because there is limited SANS data available for analysis owing to the limited number of astronauts per mission. The utilization of transfer learning can enhance the proficiency and efficacy of deep learning models in instances when the data may be limited or when training deep neural networks from scratch is computationally challenging [104,105]. A pre-trained model refers to a widely recognized model that has undergone training on a vast dataset, such as VGG16 or ResNet [105,106]. Its primary function is to extract high-level features from the original data. The act of extracting advanced features is commonly referred to as feature extraction. Subsequently, those already extracted features are utilized as input for a novel model, which is trained on a reduced dataset, featuring a lower number of parameters to be optimized [104,107]. The act of refining a pre-existing model using a reduced dataset is commonly referred to as fine-tuning [105,106]. Although it is a powerful technique, the validity of this technology to address limited SANS datasets must be rigorously tested prior to deployment.

Transfer learning has the potential to enhance the efficiency of AI systems in detecting ocular abnormalities in-line with SANS, in cases where medical data is limited. The lack of medical supplies and imaging modalities in space implies that to achieve thorough and effective visual examination, low mass and low footprint equipment must be utilized. Transfer learning techniques can be utilized to train a deep neural network for the purpose of detecting SANS. This can be achieved by utilizing pre-trained models that were originally designed for image classification tasks, such as ImageNet.

In the context of spaceflight and Earth applications for the detection of ocular disorders, the use of transfer learning provides numerous benefits:

- Addressing Data Scarcity: Space missions often have limited resources, including medical data. Transfer learning helps AI systems overcome the problem of sparse data by using prior knowledge from large, publicly accessible datasets such as ImageNet.
- Effective Model Training: The amount of computing power and training time are reduced when pre-trained models are fine-tuned using a limited dataset of retinal pictures acquired in space. In resource-constrained areas such as space, this effectiveness is important. Furthermore, transfer learning combined with pre-trained models allows AI systems to identify ocular anomalies in astronauts with high accuracy, assisting in the early detection and treatment of SANS-related problems.

Fine-tuning pre-trained models on a small dataset of retinal images obtained in space may serve as a promising solution to address the issue of limited data [105].

The application of transfer learning in machine learning involves training a model on a vast dataset to acquire comprehensive features, which are subsequently utilized to train another model on a smaller dataset [108]. The concept involves utilizing the insights acquired from the extensive dataset to enhance the efficacy of the model on the comparatively smaller dataset. The aforementioned circumstance holds significant value, especially when the smaller dataset is constrained, which is a common occurrence in medical imaging domains, encompassing those pertaining to SANS.

Fine-tuning refers to the practice of enhancing the performance of a pre-existing model by training it on a smaller dataset [79]. This process involves utilizing the knowledge gained from the pre-training phase to further optimize the model’s performance. There are two methods for performing fine-tuning on a pre-trained model. The first involves the freezing of pre-existing layers and the addition of new layers atop the model. The second method entails the unfreezing of select layers within the pre-trained model, followed by the retraining of the entire model [79].

An example of fine-tuning involves the utilization of a pre-trained model on a vast dataset of retinal fundus images, which can be further optimized by adapting to a smaller dataset of retinal images obtained during a space mission [79]. The proposed approach would enable a precise and resilient examination of the images while minimizing the need for substantial additional data. Similarly, it is feasible to adjust a pre-existing model that has undergone training on an extensive range of OCT images by utilizing a smaller dataset of OCT images that have been gathered during a space mission. The adoption of this methodology would facilitate an accurate and robust analysis of the images, without requiring a substantial amount of new information [79].

The utilization of transfer learning can prove to be advantageous in the field of SANS due to the lack of extensive datasets that are required for the training of supervised and unsupervised models [37]. The technique of transfer learning enables the adaptation and refinement of models that have been trained on extensive datasets to operate effectively on relatively smaller datasets [37]. Implementing this model during spaceflight and for the assessment and diagnosis of SANS can offer significant benefits in situations where data collection is constrained due to the logistical complexities associated with performing medical imaging procedures in space and/or the sharing of medical images from Earth to the ISS.

## 9. Limitations of the Application of Artificial Intelligence to SANS

There are various limitations to the AI technology mentioned above for SANS. A primary limitation is the validation of the technology. As there is already a limitation in training data for SANS, there will be a similar difficulty in understanding whether such AI technology can provide robust outcomes on real SANS data. GAN technology to help synthesize SANS images may help with datasets; however, true accuracy data must depend on real SANS images. Another limitation is the computational and electronic capability to facilitate such advanced techniques over an extended period of time. The computational and processing requirements to facilitate such techniques may induce a relative strain of resources for spacecraft and space travel where many resources are limited. Although these technologies are helpful for SANS, careful consideration must be placed on the resources such technologies take over the course of prolonged missions.

## 10. Future Direction and Conclusions

AI has revolutionized how we are approaching terrestrial ophthalmic diseases. These same techniques can be applied to ophthalmic imaging. Current AI research for SANS is focused on building architectures for the detection of SANS and further understanding the pathophysiology of this neuro-ophthalmic phenomenon for orbital and planetary travel [37]. Research is also focused on merging extended reality technology for SANS and other ophthalmic conditions during spaceflight, which can be merged with AI techniques [50,109,109,110,111,112,113]. Additionally, as these AI models are built for austere environments, these emerging and revolutionary techniques can also be applied to areas on Earth with limited medical capabilities and resources [37]. Another utilization of AI for SANS may be the combination of genetic and nutritional data with imaging data to improve personalization medicine in SANS. As we look towards the Mars mission and other planetary missions, the various advances in AI applications can help to further understand SANS and monitor this neuro-ophthalmic phenomenon.

## Figures and Tables

**Table 1:** Hypotheses for the pathogenesis of spaceflight-associated neuro-ocular syndrome (SANS).

Hypothesis	Summary	Reference
Cephalad fluid shift and elevated intracranial pressure	Drops in hydrostatic pressure cause widespread disruption of cranial fluids. These shifts can cause venous congestion and block cerebrospinal outflow, resulting in excess pressure on the optic nerve sheath (ONS). Congestion in the cranial vascular system and CSF spaces causes ICP to increase. The elevation in overall pressure is transferred to the ocular system via the ONS, which may cause SANS findings.	Mader et al., 2011 [5] Marshall-Goebel et al., 2019 [12] Orešcović & Bulat, 1993 [13] Martin Paez et al., 2020 [33] Zhang et al., 2018 [9] Zhang et al., 2014 [34] Lee et al., 2020 [1]
Brain upward shift	Microgravity can cause the brain to shift upwards, retracting the optic nerve and compressing CSF spaces. Reduced CSF space volume results in congestion and a subsequent increase in CSF pressure.	Roberts et al., 2017 [22] Shinojima et al., 2018 [23]
Ocular glymphatic dysfunction	The ocular glymphatic system is an offshoot of the general glymphatic system, a series of perivascular spaces (PVS) that transport CSF. Altered venous and arterial flows modify these PVS; such changes both increase CSF inflow and impair its outflow and drainage, causing CSF buildup near and along the ONS.	Jessen et al., 2015 [24] Mathieu et al., 2017 [26] Wang et al., 2020 [27] Wostyn et al., 2018 [28] Wostyn et al., 2022 [25]
Genetics	Several polymorphisms within 1-carbon metabolites have been correlated with increased presentation of SANS symptoms, suggesting that genetic mechanisms could predispose astronauts to developing the syndrome.	Zwart et al., 2012 [31] Zwart et al., 2016 [29] Zwart et al., 2019 [30]
Vitamin B levels	Higher levels of vitamin B have been correlated with lower incidence and magnitude of SANS symptom development.	Zwart et al., 2016 [29] Zwart et al., 2019 [30]
Reduction of tissue compressive forces	Reduction of tissue compressive forces in microgravity may lead to a reduction in transmural pressure at the posterior aspect of the eye. Persistent transmural reduction may lead to ocular remodeling and SANS.	Buckey et al., 2022 [16] Buckey et al., 2018 [19] Norsk et al., 2020 [18]
Cerebral blood volume pulsatility	Cerebral blood volume pulsatility may be increased during spaceflight. This persistent increase in pulsatility during long-duration spaceflight may affect nearby ocular structures to cause remodeling.	Strangman et al., 2017 [21]

## Data Availability

No new data were created or analyzed in this study. Data sharing is not applicable to this article.
